# IridiumRF-MR: A Multi-Receiver One-Dimensional I/Q Dataset for Specific Emitter Identification of LEO Satellites

**DOI:** 10.3390/s26206418

**Published:** 2026-10-11

**Authors:** Jiarong Duan, Yuhang Zheng, Bo Zang, Ke Wang, Meng Jia, Yi Zhang

**Affiliations:** 1 School of Electronic Engineering, Xidian University, Xi’an 710071, China; 2 School of Computer Science and Engineering, Xi’an University of Technology, Xi’an 710048, China

**Keywords:** specific emitter identification, multi-receiver, one-dimensional I/Q dataset, Iridium downlink signals, radio frequency fingerprinting

## Abstract

Aiming at the lack of publicly available datasets of satellite Specific Emitter Identification (SEI) and the problem of insufficient data under multi-receiver conditions, this paper constructs a multi-receiver one-dimensional I/Q dataset for Iridium downlink signals using six ADALM-PLUTO software-defined radio receivers. The dataset contains a total of 367,658 receiver-level valid signal observations covering 66 Iridium satellites. Each sample contains metadata such as SNR, acquisition timestamp, location information, and environmental information. The dataset preserves the unmodulated tone, BPSK field, and QPSK data segment of each detected Iridium burst as one-dimensional I/Q sequences, while the multi-receiver acquisition configuration enables the investigation of receiver-dependent effects under common observation conditions. Representative classification experiments with multiple deep-learning models, different input lengths, different signal-to-noise ratios, and cross-receiver evaluations are provided as technical validation to demonstrate the usability of the released data for downstream satellite specific emitter identification tasks. The dataset provides a publicly available data resource for satellite radio frequency fingerprinting, cross-receiver identification, and low-SNR signal studies.

**Dataset:** The IridiumRF-MR dataset is publicly available in Zenodo at DOI: 10.5281/zenodo.21926940.

**Dataset License:** CC BY 4.0

## 1. Introduction

In recent years, the construction of global large-scale constellation networks has been accelerating. Constellations such as Starlink, OneWeb, and Kuiper, as well as China’s Star Network and Qianfan Constellation, are building a satellite internet with low latency, high bandwidth, and global coverage, and have become an important part of the new generation of information infrastructure [1]. However, as the construction of satellite internet enters the era of large-scale and standardized development, the space electromagnetic environment is facing severe security threats. The wireless propagation characteristics of the satellite-to-ground link and the inter-satellite link make satellites extremely vulnerable to attacks such as deception, replay, signal interference, and illegal access [2]. Relying solely on traditional authentication mechanisms is insufficient to defend against covert attacks at the physical layer and link layer. Therefore, it is of significant research value to supplement lightweight and non-cryptographic identity authentication methods at the physical layer for satellites.

Specific emitter identification (SEI) distinguishes transmitters through subtle and difficult-to-clone radio-frequency (RF) fingerprints introduced by hardware non-idealities in oscillators, mixers, power amplifiers, filters, and other components [3]. In actual scenarios, satellite signals often encounter problems such as low signal-to-noise ratio and significant Doppler frequency shift. They are also affected by complex factors such as the atmosphere, ionosphere scintillation, and receiver chromatic aberration, resulting in a significant decline in the performance of traditional radiation source individual identification technologies. With the development of artificial intelligence, deep learning has been introduced into the field of individual identification of radiation sources. Deep neural networks can learn fingerprint features from the data, but their effectiveness largely depends on the quality, scale, diversity, reliability of annotation, and accessibility of the underlying datasets.

Beyond specific emitter identification, non-cooperative LEO satellite signals of opportunity have also been exploited for passive navigation and positioning. Tan et al. investigated positioning using Iridium satellite signals of opportunity, demonstrating the potential of Iridium downlink signals for opportunistic localization [4]. More recently, Florio et al. investigated LEO-based coarse positioning through angle-of-arrival estimation of signals of opportunity, further demonstrating the applicability of passively received LEO signals to localization and sensing tasks [5]. These studies indicate that LEO satellite signals have broader potential for passive sensing and exploitation beyond specific emitter identification.

Publicly documented datasets for Iridium satellite fingerprinting remain limited. PAST-AI uses a single receiver to construct a benchmark image dataset covering 66 satellites of the Iridium constellation, and uses the open-source tool Iridium-Toolkit to parse the demodulated DQPSK broadcast data packets to obtain plaintext satellite hardware identification as the real label [6]. However, the method of obtaining labels through analysis and demodulation has limitations in non-cooperative scenarios, and the data from a single receiving device cannot capture the differences between the receiving ends, making it difficult to meet the requirements of cross-receiver identification.

To address these limitations, this paper presents IridiumRF-MR, a multi-receiver one-dimensional I/Q dataset for individual identification of Iridium satellites. Six ADALM-PLUTO SDR receivers were deployed at the same receiving site to acquire Iridium downlink signals in parallel under common observation conditions. In IridiumRF-MR, six ADALM-PLUTO receivers are connected to a common antenna path through a one-to-six power divider. This controlled acquisition configuration is designed to maintain, as far as possible, consistent satellite signals, propagation conditions, antenna characteristics, and common RF front-end conditions across the six receiving chains. The resulting receiver-specific observations can therefore be compared to investigate receiver-dependent variations introduced by the physically independent SDR receiving chains.

Satellite identities were assigned by matching the measured Doppler frequency shifts and acquisition timestamps with theoretical Doppler predictions derived from TLE data and STK-based orbital calculations. The dataset covers all 66 Iridium satellite classes and contains 367,658 receiver-level valid samples. Each sample is associated with metadata including SNR, acquisition timestamp, receiving-site coordinates, and environmental information. The main contributions are as follows:

- A multi-receiver one-dimensional I/Q dataset covering 66 Iridium satellites is constructed using six physically independent ADALM-PLUTO receivers connected to a common antenna through a one-to-six power divider. This controlled acquisition design maintains consistent satellite signals, propagation conditions, antenna characteristics, and common RF front-end conditions across the six receiving chains, thereby enabling the investigation of receiver-dependent variations through comparisons of receiver-specific observations;
- The satellite label is annotated by matching the minimum error between the measured Doppler frequency offset and the theoretical Doppler frequency offset, which improves the applicability of the data annotation method in non-cooperative scenarios;
- Technical validation is provided through satellite identification experiments involving multiple model architectures, different input-sequence lengths, different SNR conditions, and cross-receiver evaluation to demonstrate the usability of the released dataset for downstream RF-fingerprinting studies.

## 2. Related Dataset

Currently, the publicly available dataset that is closest to this paper is PAST-AI. This dataset covers 66 satellites in the Iridium constellation and uses the open-source tool iridium-toolkit to demodulate the DQPSK broadcast data packets. It generates real labels by obtaining the plaintext satellite hardware identifiers. The continuous I/Q sampled data is mapped to the complex plane and converted into grayscale images, which are used for training and testing deep learning models [6]. PAST-AI provides an important benchmark for satellite physical-layer identity authentication research, but its data is collected by a single receiver, making it difficult to represent the hardware differences between different receiving devices. Moreover, the label acquisition relies on demodulating the broadcast data packets, and its applicability in non-cooperative scenarios is limited.

More recently, Solenthaler et al. introduced OrbID, an RF-fingerprinting dataset for the Orbcomm satellite constellation collected using different SDRs and physical locations [7]. OrbID demonstrates the feasibility of combining observations acquired by different SDR devices at geographically distributed receiving sites, thereby incorporating both receiver and location-dependent diversity into the collected data.

The IridiumRF-MR dataset constructed in this paper differs from these existing datasets. The dataset retains the one-dimensional time-domain I/Q sequence instead of only using graphical representation, which is conducive to directly conducting research on the extraction of original signal features. A distinctive aspect of IridiumRF-MR is its controlled co-located multi-receiver acquisition architecture. Six physically independent ADALM-PLUTO receivers are supplied with the same filtered and amplified antenna signal through a one-to-six power divider. Thus, unlike geographically distributed multi-receiver datasets, IridiumRF-MR is not intended to characterize spatial diversity or location-dependent propagation effects. Instead, its design intentionally controls the antenna, observation location, and common propagation conditions so that receiver-to-receiver differences can be studied with reduced confounding from these factors.

## 3. Dataset Description

### 3.1. Dataset Overview

**Figure 1:**
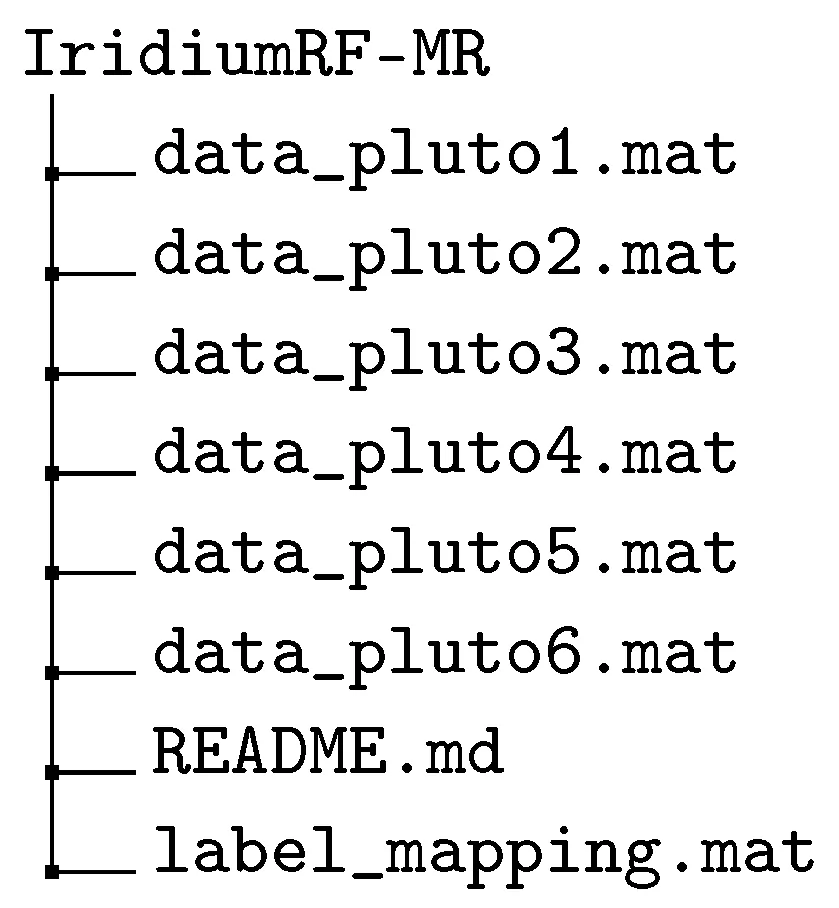
Organization of the IridiumRF-MR dataset.

The IridiumRF-MR dataset is released in the MATLAB MAT file format. Its file organization structure is shown in Figure 1, and it includes six corresponding .mat data files for the receivers and two auxiliary instruction files. README.md is used to explain the overall structure of the dataset, including the naming rules of each data file, variable meanings, array dimensions, data types, physical units, I/Q channel arrangement method, and MATLAB (version R2021a) reading methods, to facilitate users to correctly parse and use the data. label_mapping.mat is used to record the one-to-one correspondence between digital category labels and Iridium satellite identities, mapping the 0–65 digital labels in satlabel to the corresponding satellite name numbers to ensure that the six receiver data files adopt consistent category definitions.

The measurements were collected in Xi’an, Shaanxi Province, China, at approximately 108.9139° E and 34.2361° N. Acquisition lasted approximately 72 h and covered multiple satellite passes. Six ADALM-PLUTO SDR receivers (Analog Devices, Inc., Wilmington, MA, USA) captured the same filtered and amplified antenna signal through a one-to-six power divider. The use of a shared antenna and common RF distribution path was intentional. Rather than introducing spatial diversity, this configuration was designed to provide comparable RF excitation to six physically independent receiver chains. This reduces variations associated with different antennas, receiving locations, and propagation paths and allows receiver-dependent effects to be examined under controlled observation conditions. Each receiver observed all 66 Iridium classes. After common preprocessing and quality screening, the dataset contained 367,658 receiver-level valid samples. The reported 367,658 samples represent receiver-level observations rather than 367,658 independent over-the-air satellite transmissions. Because the six receivers are supplied by a common antenna signal through the one-to-six power divider, the same physical Iridium burst may be observed by more than one receiver.

Each receiver-specific MAT-file adopts the same variable naming convention and data organization to ensure consistency across the six receiving devices. The stored variables include three types of one-dimensional I/Q signal segments, namely the unmodulated tone, BPSK field, and QPSK data segment, together with satellite identity labels, measured Doppler shifts, signal-to-noise ratios, acquisition timestamps, receiving-site coordinates, and environmental information. The signal arrays and their associated metadata are aligned by sample index, so that each I/Q sample can be directly associated with its corresponding satellite identity and acquisition conditions. Table 1 summarizes the content, array dimensions, data types, and physical units of the variables stored in each MAT-file. The dimensions listed in the table correspond to data_pluto1.mat, which contains 63,407 valid samples; the other receiver files follow the same structure but contain different numbers of valid samples.

### 3.2. Iridium Signal Structure

Iridium signals are transmitted in the L band, and the user link employs a hybrid multiplexing scheme combining spatial division multiple access (SDMA), frequency division multiple access (FDMA), time division multiple access (TDMA), and time division duplexing (TDD). The overall operating band extends from 1616.0 to 1626.5MHz. The 1616.0–1626.0MHz band is allocated to duplex channels for bidirectional service-data transmission. This 10MHz duplex band is divided into 30 sub-bands, each containing eight channels. Each sub-band has a bandwidth of approximately 333.333kHz, while each channel occupies approximately 41.67kHz. The 1626.0–1626.5MHz band is allocated to simplex channels for signaling and broadcast-message transmission. This 0.5MHz simplex band is divided into 12 channels, each with a bandwidth of approximately 41.67kHz. Among the 12 channels of the Iridium simplex downlink user link, persistent burst signals are mainly observed on channels 7 and 11. Channel 7 carries Ring Alert messages and has a nominal center frequency of 1626.270833MHz, whereas channel 11 carries Primary Message traffic and has a nominal center frequency of 1626.43750MHz. A complete Iridium TDMA frame has a duration of 90ms. The baseband symbol rate is 25ksymbols/s, corresponding to 2250 symbols per frame. Within each 90ms frame, the nominal duration of the downlink simplex time slot is 20.32ms, whereas the actual duration of simplex-channel signals on the Iridium downlink user link ranges from 6.5 to 20.32ms. The simplex time slot is followed by four uplink duplex time slots and four downlink duplex time slots, with each duplex time slot lasting approximately 8.28ms [8].

A complete Iridium downlink TDMA frame has a duration of 90ms. For each frame of signal, the burst signal consists of three parts: the unmodulated sinusoidal single-tone signal, the BPSK modulated independent data field, and the QPSK modulated data information field.

### 3.3. Class Labels and Receiver Statistics

Table 2 presents the number of valid samples retained by the six receivers and the corresponding number of satellite classes. All six receivers provide complete coverage of the 66 Iridium satellites. Owing to variations in signal-to-noise ratio, observation elevation angle, and burst-detection success across different satellite passes, the number of valid samples differs slightly among the receivers; however, no satellite classes are missing.

**Figure 2:**
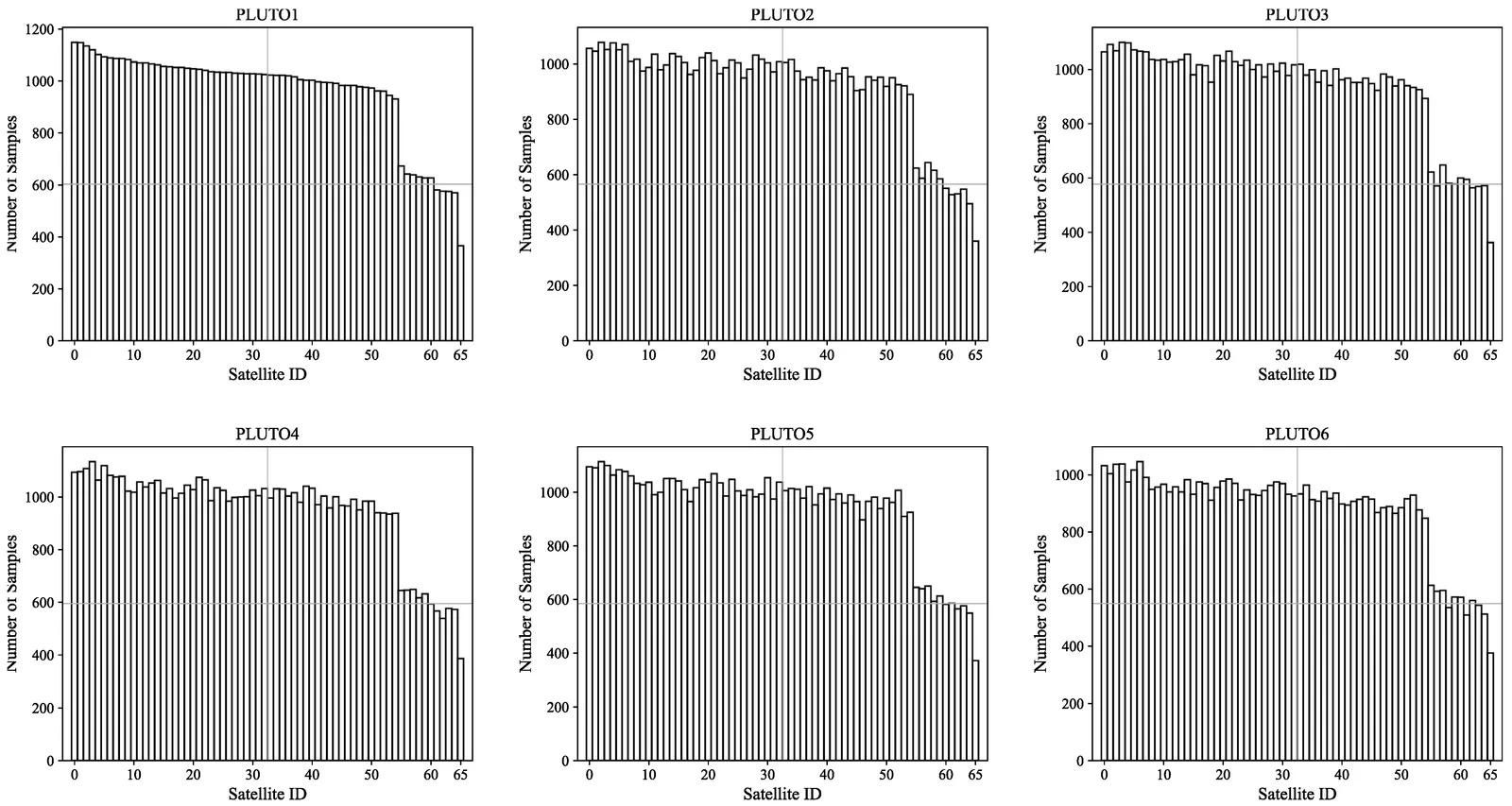
The distribution of samples from each receiver among different satellite categories. Satellite labels range from 0 to 65. The gray horizontal lines are plotting gridlines provided for visual reference only.

Figure 2 further illustrates the per-satellite sample distribution for the six receivers. Overall, the class distributions are highly consistent across the receivers, with most satellites contributing approximately 800–1100 samples and exhibiting a relatively balanced distribution. Only a small number of satellites contain fewer valid samples because of unfavorable observation conditions, such as low-elevation angles and degraded signal quality during their passes. Consequently, IridiumRF-MR exhibits consistent data scale, class coverage, and sample distribution across different receivers, providing a stable data basis for subsequent cross-receiver satellite identification and model performance evaluation.

**Figure 3:**
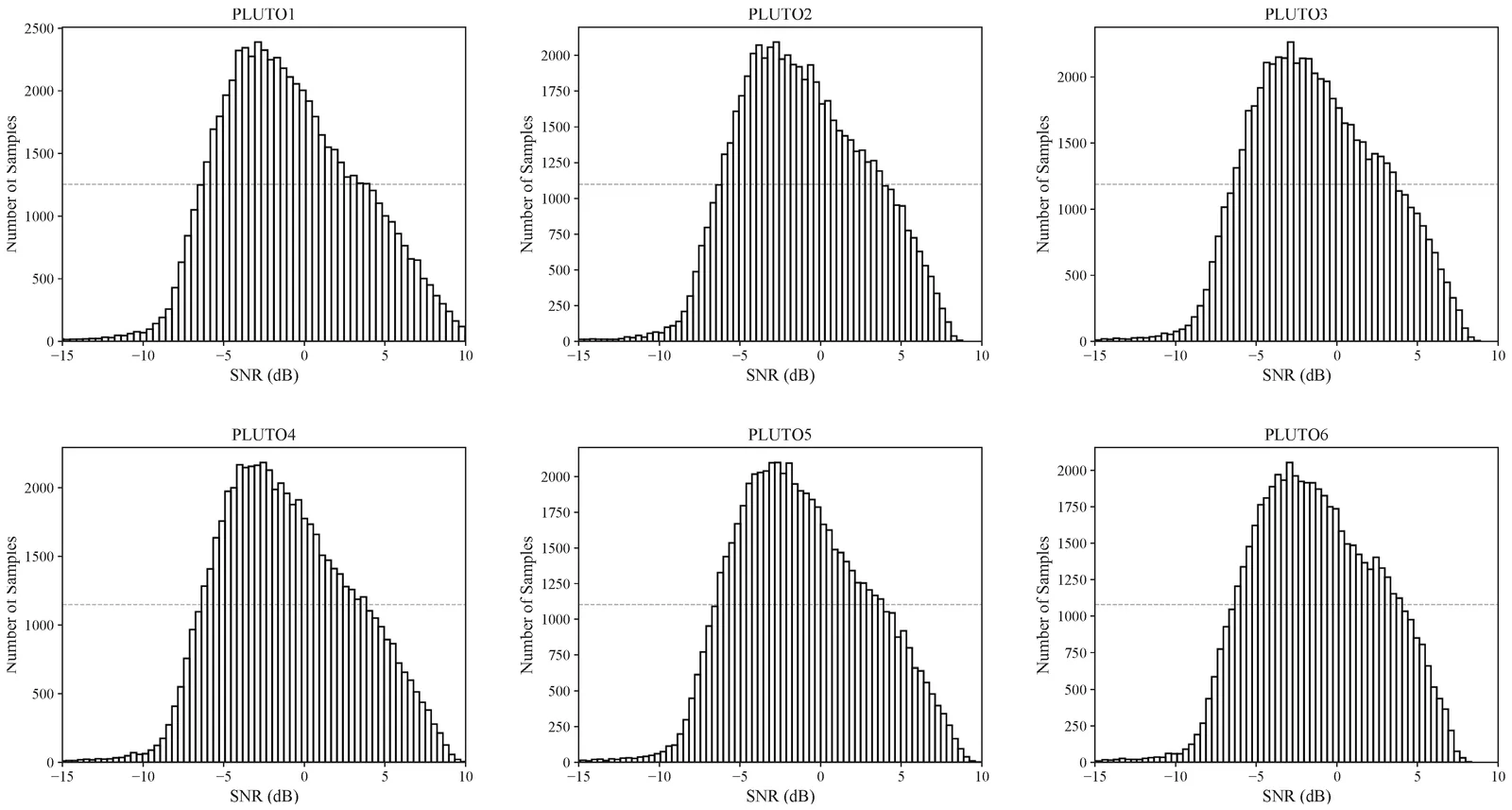
Distribution of valid samples across SNR intervals for the six receivers.

Figure 3 shows the distribution of samples from different receivers across different SNR intervals. Most samples are concentrated in the range of −15 to 10dB, indicating that the dataset can not only support individual identification of Iridium satellites, but also be used to verify and evaluate model recognition performance under low-SNR conditions.

## 4. Methods

### 4.1. Hardware Acquisition System

**Figure 4:**
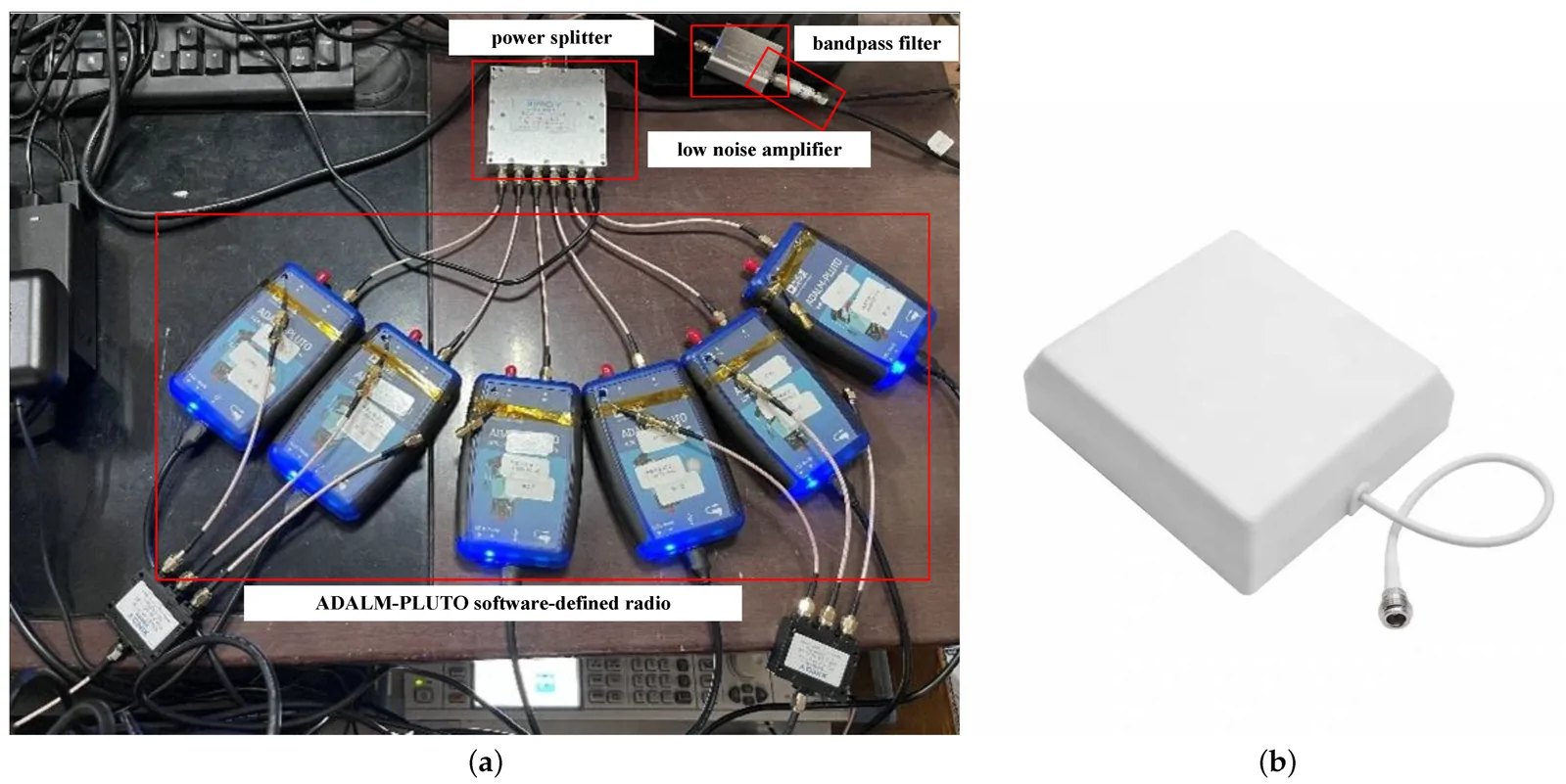
Hardware acquisition system: (**a**) six ADALM-PLUTO SDR receivers connected to the outputs of the one-to-six power divider; (**b**) directional flat-panel antenna.

Data collection was carried out in Xi’an, Shaanxi Province, with a collection duration of about 72 h, covering multiple Iridium satellite overhead Windows, and the timestamp of each valid signal sample was automatically recorded during the collection process, which was used for subsequent matching with satellite orbit information. The hardware acquisition system is shown in Figure 4a, and the overall link is mainly composed of five parts cascaded: antenna (Figure 4b), low-noise amplifier, bandpass filter, power splitter and software radio platform. The antenna covered approximately 800 MHz–3700 MHz. The LNA operated over approximately 100 kHz to 6 GHz with a typical gain of 30 dB. The band-pass filter covered the Iridium band from 1616 MHz–1626.5 MHz. The filtered output was divided into six nominally identical RF paths.

At the data acquisition end, six ADALM-PLUTO software-defined radio (SDR) platforms based on the AD9363 RF transceiver are employed as receiving devices. Each SDR provides independent transmit and receive channels and supports an operating frequency range of 325MHz to 3.8GHz, with a configurable channel bandwidth ranging from 200kHz to 20MHz. In the receiving chain, the RF signal is sequentially processed by the integrated low-noise amplifier (LNA), mixer, analog filter, digital signal-processing module, digital filter, and finite impulse response (FIR) filter, and is subsequently digitized with a 12-bit resolution. In addition, the receive path supports both automatic and manual gain control and incorporates DC-offset and quadrature-error correction functions.

A parallel acquisition scheme for the six receivers was implemented in MATLAB. The six acquisition processes ran in parallel, with their initialization deliberately staggered at a nominal 50ms interval between adjacent receivers to mitigate USB bus contention. Accordingly, the programmed start delays ranged from 50ms for the first receiver to 300ms for the sixth. Following initialization, each receiver ran an independent continuous acquisition loop. A shared external 10MHz reference clock was fed to all six receivers to improve carrier frequency and sampling clock consistency across the receive chains.

### 4.2. Receiver Parameter Settings

Table 3 lists the principal acquisition settings. A center frequency of 1626.25 MHz and a sampling rate of 960 ksample s^−1^ were selected to cover the channel-7 downlink bursts without aliasing. The receive gain was fixed at 50 dB. Each captured frame contained 9600 complex samples, corresponding to 10 ms.

### 4.3. Signal Preprocessing

**Figure 5:**
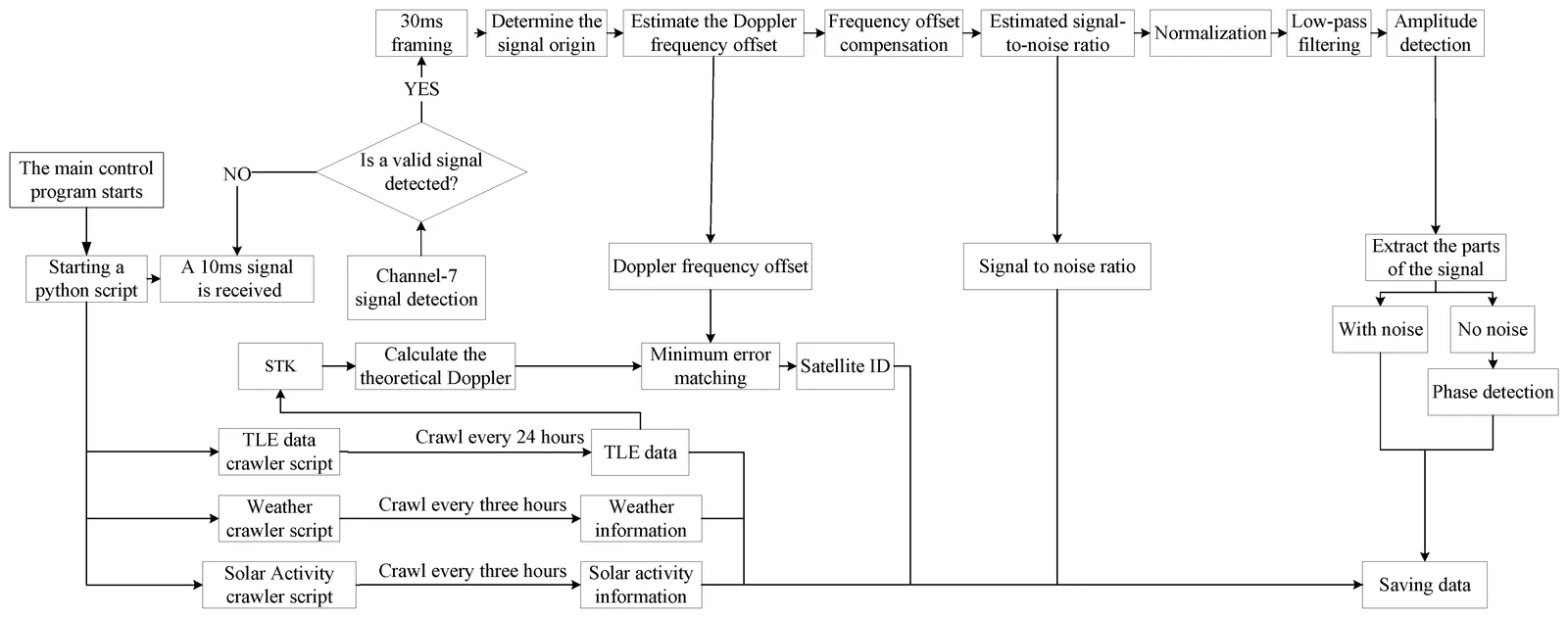
Signal preprocessing process.

The signal preprocessing procedure is illustrated in Figure 5. First, constant false alarm rate (CFAR) detection is applied to the energy of the target-channel signal to determine the onset of each burst. The false-alarm probability of the CFAR detector was set to $P_{\mathrm{FA}}=10^{-2}$. For each cell under test, 1000 reference cells were used on each side, giving a total reference window of 2000 cells. The guard-window length was set to 500 cells on each side for the initial single-frame detection and increased to 2000 cells on each side for the subsequent three-frame concatenated detection. Matched filtering was then employed to accurately locate the start and end positions of the unmodulated tone, BPSK field, and QPSK data segment. The unmodulated tone was further used to estimate the measured Doppler frequency shift through maximum-likelihood estimation, followed by frequency-offset compensation.Subsequently, amplitude normalization was performed to scale the signal amplitudes to a consistent range, and a low-pass filter was applied to further suppress residual noise and out-of-band interference. After preprocessing, the resulting data were temporarily stored as variables in the MATLAB workspace and subsequently saved in MAT-file format. Each processed sample contained the unmodulated tone, BPSK field, and QPSK data segment extracted from the same burst, together with the corresponding metadata.

The fast Fourier transform is used to implement matched filtering in the frequency domain. Let the zero-padded received sequence be x[n], the reference sequence be h[n], and the transform length be *N*. Their discrete Fourier transforms are

(1) $$X\left[k\right]=\sum_{n=0}^{N-1}x\left[n\right]e^{-j\frac{2\pi }{N}kn},\;k=0,1,\ldots ,N-1,$$

(2) $$H\left[k\right]=\sum_{n=0}^{N-1}h\left[n\right]e^{-j\frac{2\pi }{N}kn}.$$

According to the principle of matched filtering, the spectrum of the received signal is multiplied by the complex conjugate of the reference signal spectrum, resulting in the frequency-domain representation of the cross-correlation signal as follows:

(3) $$Y\left[k\right]=X\left[k\right]H^{*}\left[k\right],$$

In the formula, $H^{*}\left[k\right]$ represents the complex conjugate of H[k].

An inverse discrete N-point Fourier transform is then performed in Y[k] and mapped back to the time domain to obtain the cross-correlation output y[n].

(4) $$y\left[n\right]=\frac{1}{N}\sum_{k=0}^{N-1}Y\left[k\right]e^{j\frac{2\pi }{N}kn},\;n=0,1,\ldots ,N-1,$$

The peak index $\hat{n}=\arg\max_{n}\left|y\left[n\right]\right|$ indicates the time instant of maximum correlation between the received and reference signals, which is taken as the starting point of the valid signal.

The measured Doppler shift is estimated as follows:

(5) $${\hat{f}}_{d}=\underset{f_{d}\in \left[f_{\min},f_{\max}\right]}{\arg\max}\left|\sum_{n=0}^{N-1}x\left[n\right]e^{-j2\pi \left(f_{0}+f_{d}\right)t_{n}}\right|,$$

where $f_{0}$ is the nominal carrier frequency, $t_{n}$ is the discrete sampling time and ${\hat{f}}_{d}$ is the estimated Doppler frequency shift.

**Figure 6:**
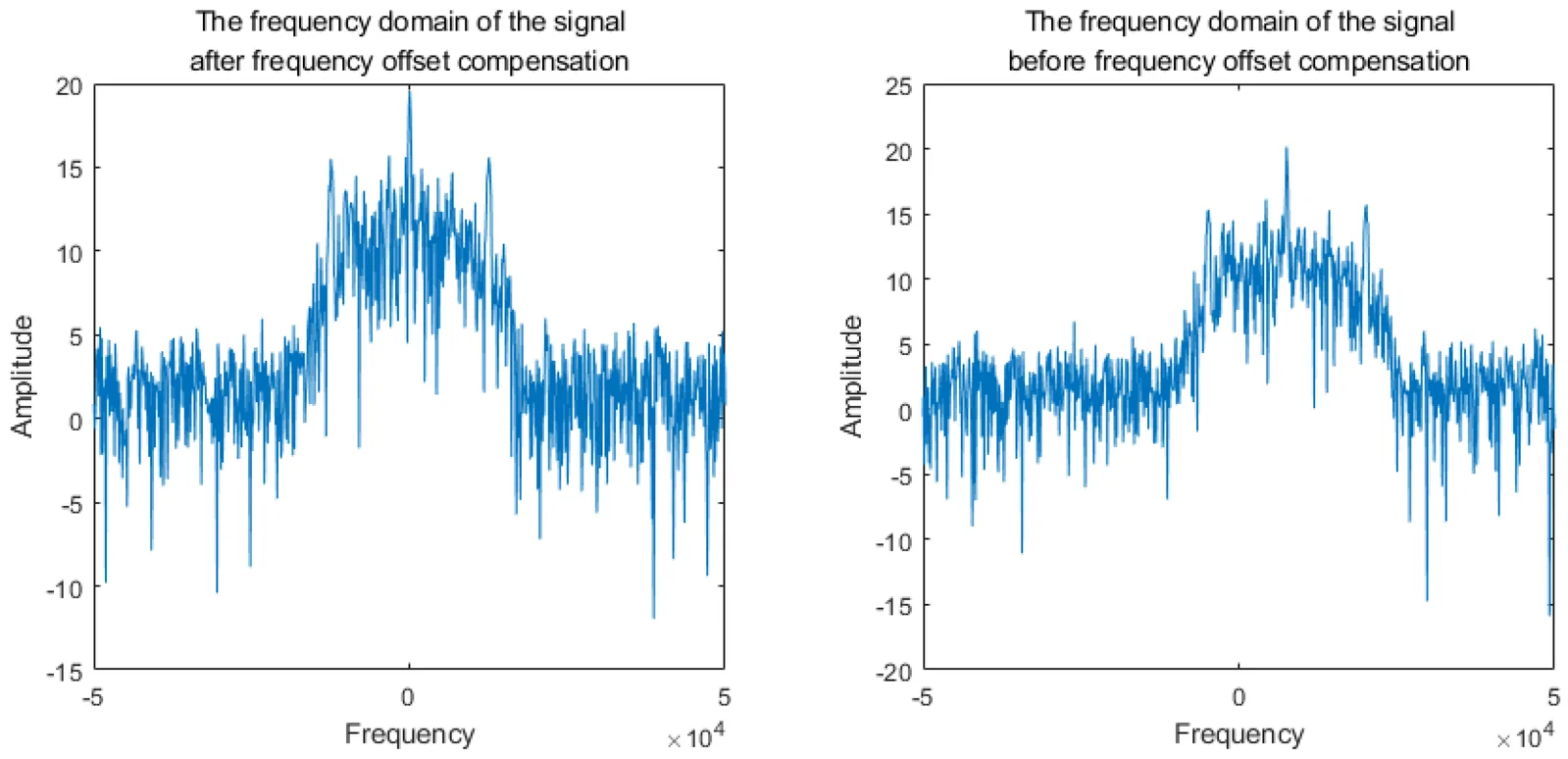
Spectrum of an example Iridium burst after and before Doppler-frequency compensation.

Figure 6 compares the spectrum of the same signal after and before frequency-offset compensation. Before compensation, the Doppler shift caused by the high-speed motion of the LEO satellite displaces the spectral center by approximately 50kHz from zero frequency. After maximum-likelihood estimation and frequency-offset compensation, the spectral center is shifted back to the vicinity of zero frequency, effectively reducing the influence of Doppler-induced frequency offset on subsequent feature extraction.

After frequency-offset compensation, amplitude normalization is performed using the maximum magnitude within the detected valid signal interval. Let $x_{c}\left[n\right]$ denote the frequency-offset-compensated complex baseband signal and S denote the detected valid signal interval. The normalized signal is expressed as follows:

(6) $$mathx_{\mathrm{norm}}\left[n\right]=\frac{x_{c}\left[n\right]}{\max_{m\in S}\left|x_{c}\left[m\right]\right|}$$

The same normalization factor is applied to both the I and Q components, preserving their relative amplitude and phase relationship.

**Figure 7:**
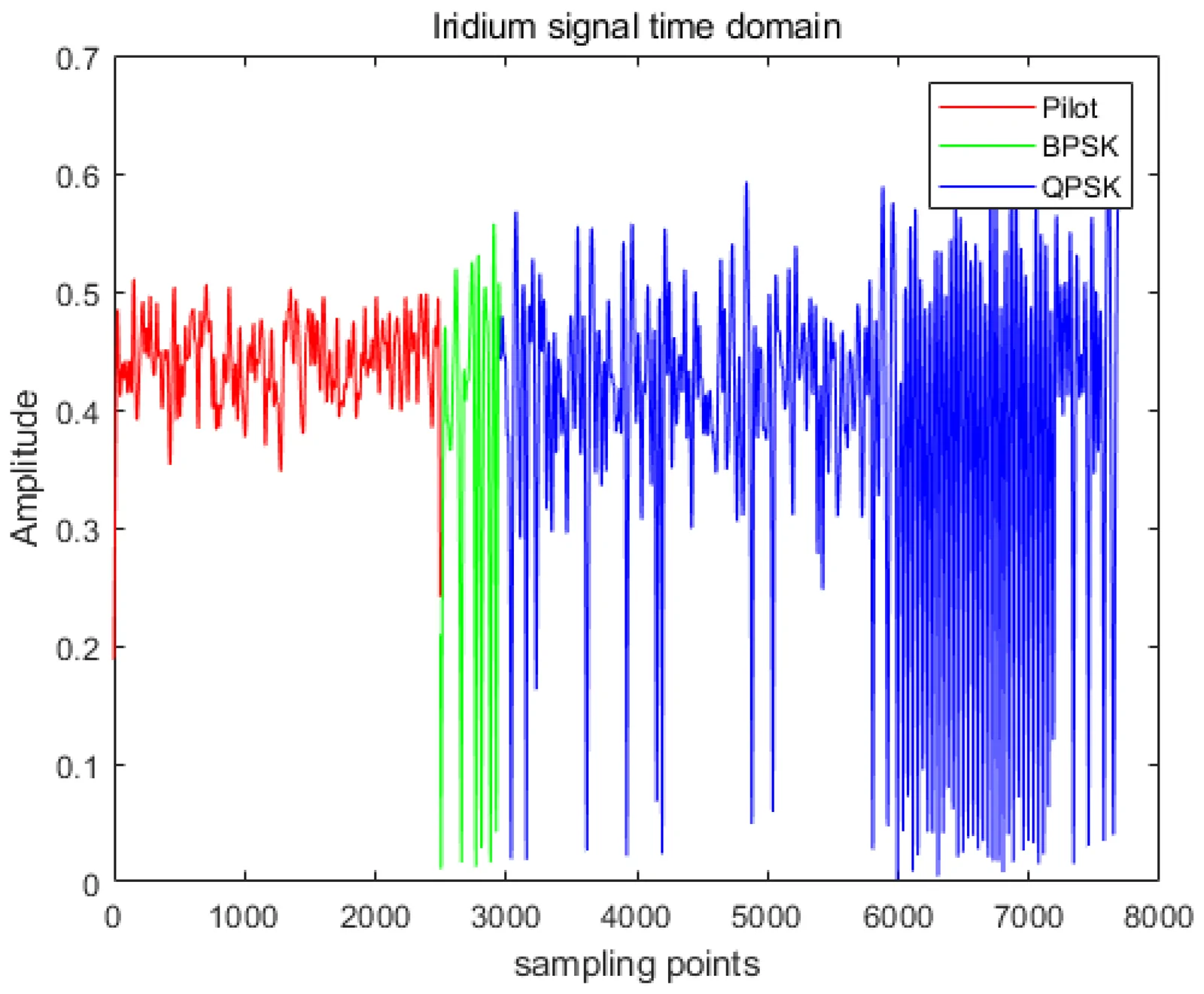
Iridium signal time-domain amplitude envelope.

Figure 7 shows the time-domain amplitude envelope of an Iridium burst after frequency-offset compensation and low-pass filtering. The signal region exhibits a clear increase in amplitude relative to the noise floor, forming a distinct raised envelope. The beginning and end of this envelope correspond to the arrival and disappearance of the valid burst signal, respectively.

**Figure 8:**
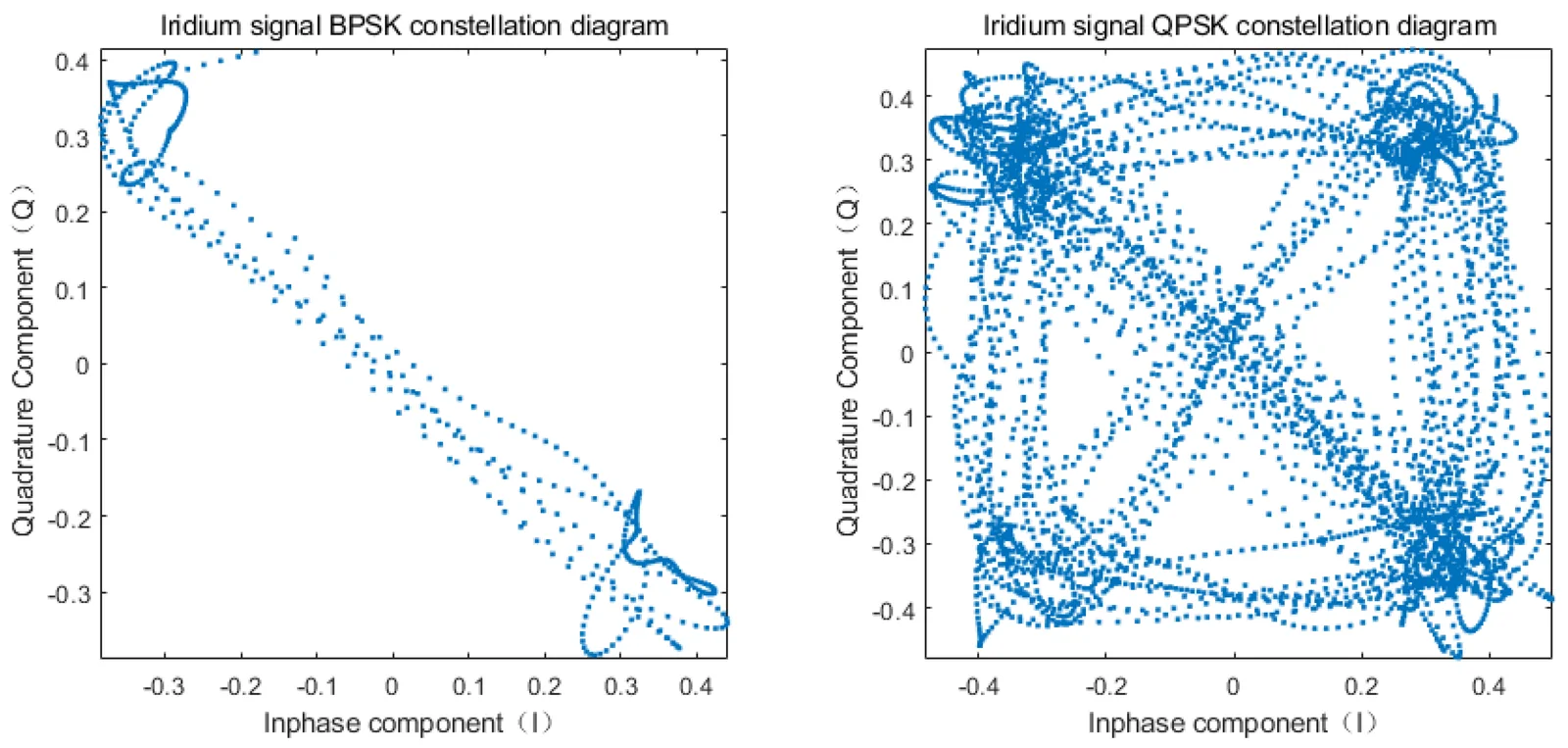
BPSK field and QPSK data segment constellation diagram.

Figure 8 shows the constellation diagrams of the BPSK field and QPSK data segment after frequency-offset compensation and low-pass filtering. The BPSK constellation exhibits two clearly separated clusters corresponding to two phase states. The large inter-cluster separation and compact intra-cluster distribution indicate good signal quality. The QPSK constellation exhibits four approximately orthogonal clusters, corresponding to four distinct phase states.

### 4.4. Satellite Identity Annotation

For each signal sample, the acquisition time was first used to determine the set of candidate Iridium satellites for the corresponding observation period. For each candidate satellite, its position and velocity were retrieved from STK using the TLE orbital information, and the theoretical Doppler shift was then computed from the relative radial velocity between the satellite and the ground receiving station.

Let $f_{D}^{\mathrm{mea}}$ denote the measured Doppler shift and $f_{D,s}^{\mathrm{theo}}$ denote the theoretical Doppler shift of candidate satellite *s*. The Doppler residual of each candidate was calculated as follows:

(7) $$mathe_{s}=f_{D}^{\mathrm{mea}}-f_{D,s}^{\mathrm{theo}}$$

All candidate satellites were then compared, and the satellite yielding the minimum absolute Doppler residual was assigned as the identity label of the observed signal:

(8) $$math\hat{s}=\arg\min_{s}\left|e_{s}\right|$$

For each observation, the Doppler residual of every candidate satellite was computed and retained during the annotation process, together with the theoretical Doppler shift and the residual of the selected satellite, so as to keep the matching information on which the annotation was based traceable. No fixed Doppler-residual threshold was adopted in the original annotation procedure; instead, satellite identity was determined by the relative comparison of residuals across the candidate satellites at the corresponding observation time. Because the complete Doppler-residual vector of all candidate satellites was retained in the annotation records, cases in which multiple candidate satellites exhibited similar Doppler residuals could be re-examined from these records.

To evaluate the sensitivity of the satellite-matching results to the TLE data, the matching procedure for PLUTO1 was repeated using three TLE datasets retrieved during the three-day acquisition period. Across the evaluated PLUTO1 observations, 99.9545% yielded identical satellite identities under all three TLE datasets, while only 0.0455% showed a change in the matched satellite identity. These results indicate that the Doppler-based satellite-matching procedure for PLUTO1 is highly stable with respect to the TLE data used within the approximately 72-h acquisition period.

Satellite classes are represented by integers from 0 to 65. Table 4 gives an excerpt of the label-to-satellite mapping.

### 4.5. Signal-to-Noise Ratio Estimation

The power estimation method based on frequency-domain energy separation is adopted to calculate the signal-to-noise ratio for each full segment of the valid sample signal. The time-domain signal is transformed to the frequency domain through the Fast Fourier Transform and the total receiving power of the full frequency band is calculated. To distinguish the signal from the noise background, an effective signal bandwidth of 30 kHz centered at zero frequency is defined as the signal region. The edge frequency band data outside the main lobe area and without signal coverage at both ends of the spectrum is specially selected as pure noise reference. The total noise power of the full frequency band is calculated proportionally. Based on the total power of the full frequency band and the total noise power, the effective signal power is obtained. The logarithm of the ratio of the effective signal power to the total background noise power is the estimated signal-to-noise ratio:

(9) $$\mathrm{SNR}=10\log_{10}\left(\frac{P_{s}}{P_{n}}\right),$$

where $P_{s}$ and $P_{n}$ denote signal and noise power, respectively.

## 5. Technical Validation

### 5.1. Validation Design and Computational Setting

The following experiments are provided as representative use cases to technically validate the usability of IridiumRF-MR for satellite specific emitter identification. These experiments are not intended to rank individual learning architectures or to use classification accuracy as the sole measure of dataset quality. Instead, they provide reproducible baseline results illustrating how the released I/Q data can be used under different model, observation length, signal-quality, and receiver-domain conditions.

- A seven-model benchmark uses preprocessed QPSK I/Q signals of size 3000×2 to provide representative baseline results for 66-class satellite identification;
- An input-sequence-length study is conducted using signal lengths of 3000, 2048, 1024, and 512 sampling points, where the shorter sequences are randomly cropped from each sample to investigate the effect of observation-window length on radio-frequency fingerprint representation;
- An SNR experiment is performed in which the test samples are divided into consecutive SNR intervals with a step size of 2dB according to their SNR metadata, and the identification accuracy is evaluated separately within each interval;
- A single-source cross-receiver evaluation is conducted in which a model trained on PLUTO1 is directly evaluated on PLUTO2–PLUTO6 without fine-tuning to examine receiver-domain transfer under the controlled common-input acquisition configuration.

The experiments were implemented in PyTorch (version 2.13.0) and run on a desktop computer with one NVIDIA RTX 2080 SUPER GPU and 32 GB of memory (NVIDIA Corporation, Santa Clara, CA, USA). For each receiver, all valid samples were first randomly permuted without replacement using a fixed random seed and were then divided into training, validation, and test subsets with a ratio of 8:1:1. Because the partition was performed on mutually exclusive sample indices, no individual I/Q sample was shared between the training, validation, and test subsets.

### 5.2. Multi-Model Baselines

Seven representative architectures were evaluated: AlexNet [9], ResNet34 [10], a conventional CNN, CLDNN [11], CGDNN [12], VGG16 [13], and Mamba [14]. Four commonly used metrics for multiclass classification, namely, accuracy, precision, recall, and F1-score, were employed to evaluate the experimental results. Precision, recall, and F1-score were calculated using macro-averaging so that each satellite class contributed equally to the evaluation, thereby reducing the influence of class imbalance on the overall performance assessment. For sequence-based processing, the two-dimensional convolutional layers in AlexNet, ResNet34, and VGG16 were replaced by their one-dimensional counterparts, while the overall backbone structure was preserved.

For all models except Mamba, a unified set of hyperparameters was adopted to maintain consistent training settings among the conventional baseline models. The models were trained using the Adam optimizer with an initial learning rate of $1\times 10^{-4}$, a weight decay of $1\times 10^{-5}$, and a batch size of 16. Cross-entropy loss was used as the objective function. Each model was trained for 50 epochs, and the learning rate was adjusted using a StepLR scheduler.

For the Mamba model, the AdamW optimizer was employed with an initial learning rate of $1\times 10^{-4}$, a weight decay of $1\times 10^{-5}$, and a batch size of 16. The loss function was a weighted cross-entropy loss with label smoothing to account for differences in class distribution and improve generalization. An adaptive learning-rate decay strategy was used during training. In addition, early stopping and mixed-precision training were introduced to reduce overfitting and improve training efficiency.

Table 5 reports performance averaged over the six receivers.

The overall results of the multi-model comparison reveal clear performance differences among the deep learning architectures in extracting radio-frequency fingerprint features. Mamba achieved the best overall performance, with an average accuracy of 0.8567 and an F1-score of 0.8509, outperforming all baseline models. It also maintained relatively stable performance across the six receivers, indicating limited sensitivity to receiver-dependent hardware variations. This result suggests that the selective state-space mechanism of Mamba is well suited to modeling long-range dependencies in I/Q sequences, enabling the model to capture temporally distributed fingerprint characteristics associated with long-distance propagation and hardware nonlinearities while maintaining computational efficiency.

The conventional one-dimensional convolutional architectures, including VGG16, CNN, and AlexNet, achieved relatively stable overall accuracies ranging from approximately 0.80 to 0.84. ResNet34 obtained an accuracy of 0.8154 and an F1-score of 0.8077. Its residual connections facilitate information propagation through deeper layers and alleviate optimization difficulties associated with deep networks, contributing to relatively robust performance across different receivers. In contrast, the hybrid architectures CLDNN and CGDNN exhibited lower overall performance and greater variation across the six receivers. These results suggest that, under complex and low-SNR channel conditions, combining multiple heterogeneous feature-extraction modules does not necessarily improve radio-frequency fingerprint representation and may increase model sensitivity to receiver- and channel-dependent variations.

**Figure 9:**
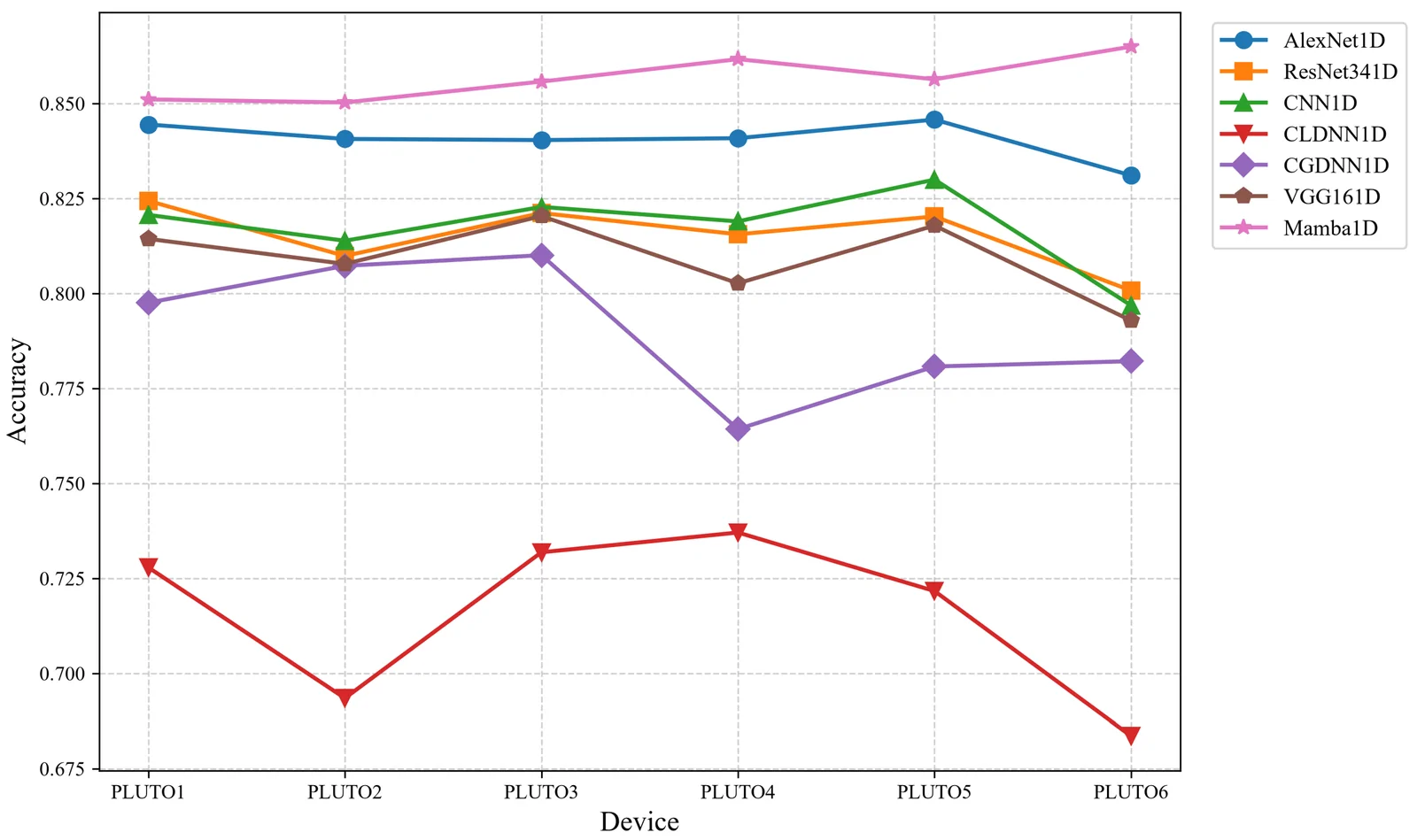
The recognition accuracy rates of the seven benchmark models on different receivers.

**Figure 10:**
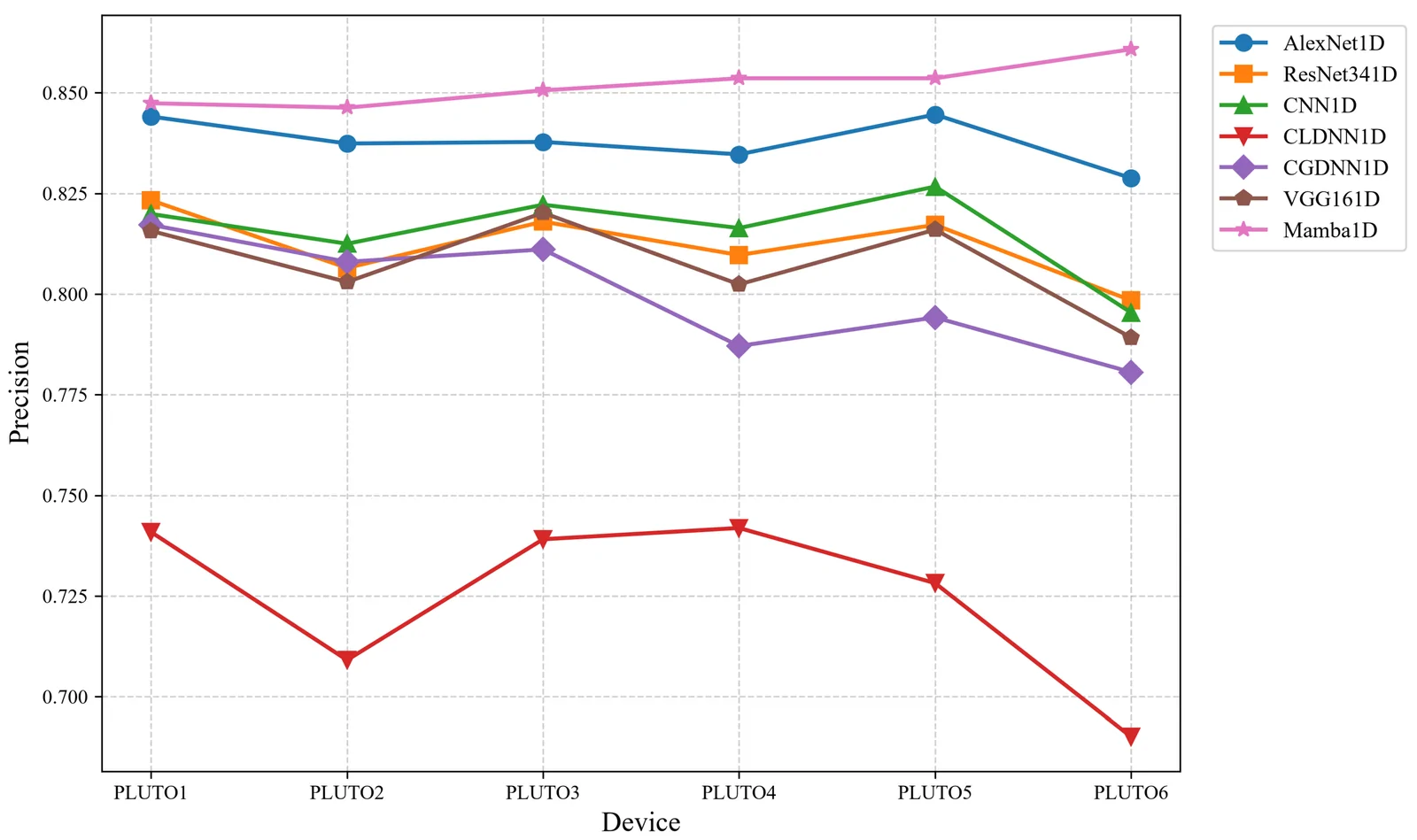
The recognition precision rates of the seven benchmark models on different receivers.

**Figure 11:**
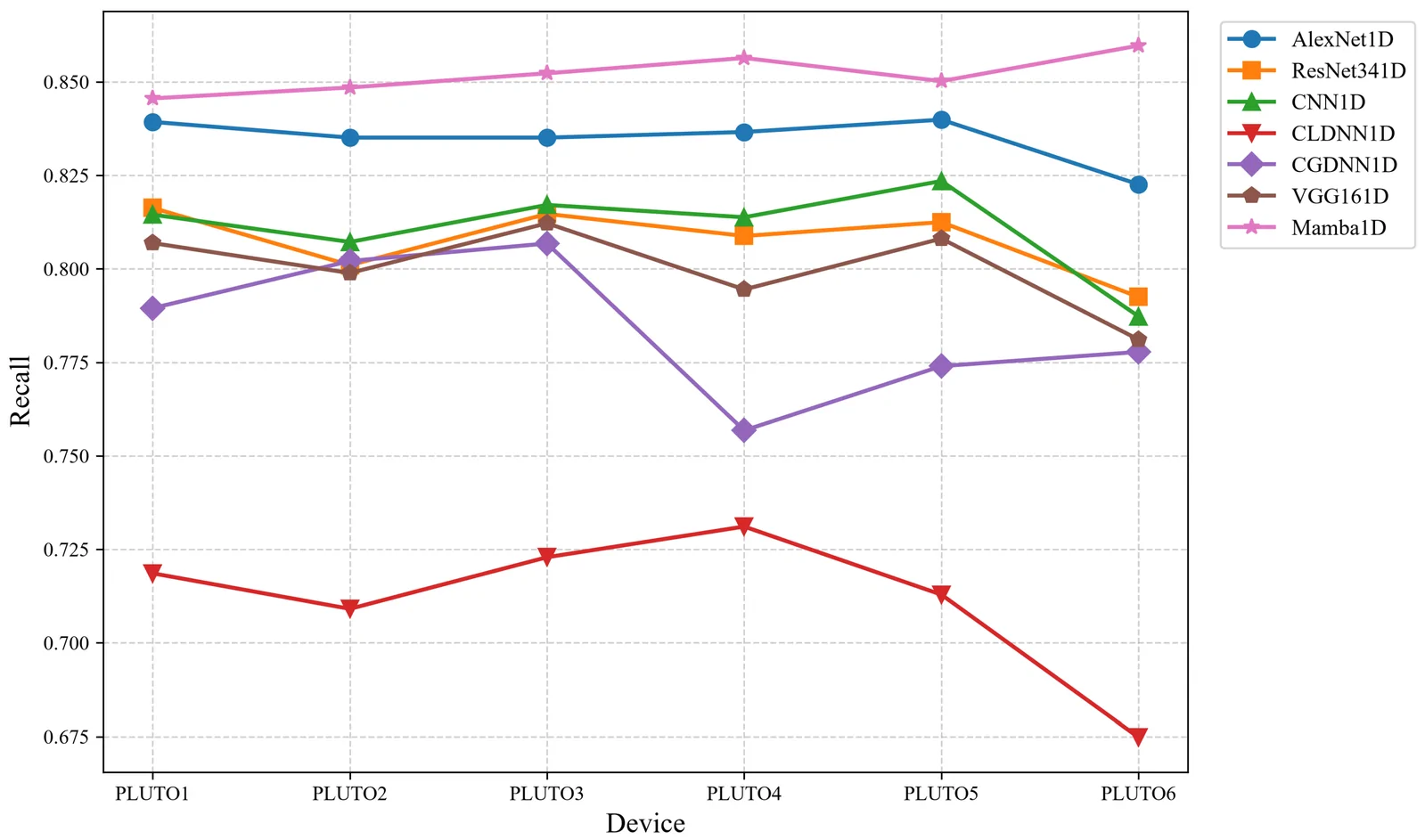
The recall rates of the seven benchmark models on different receivers.

**Figure 12:**
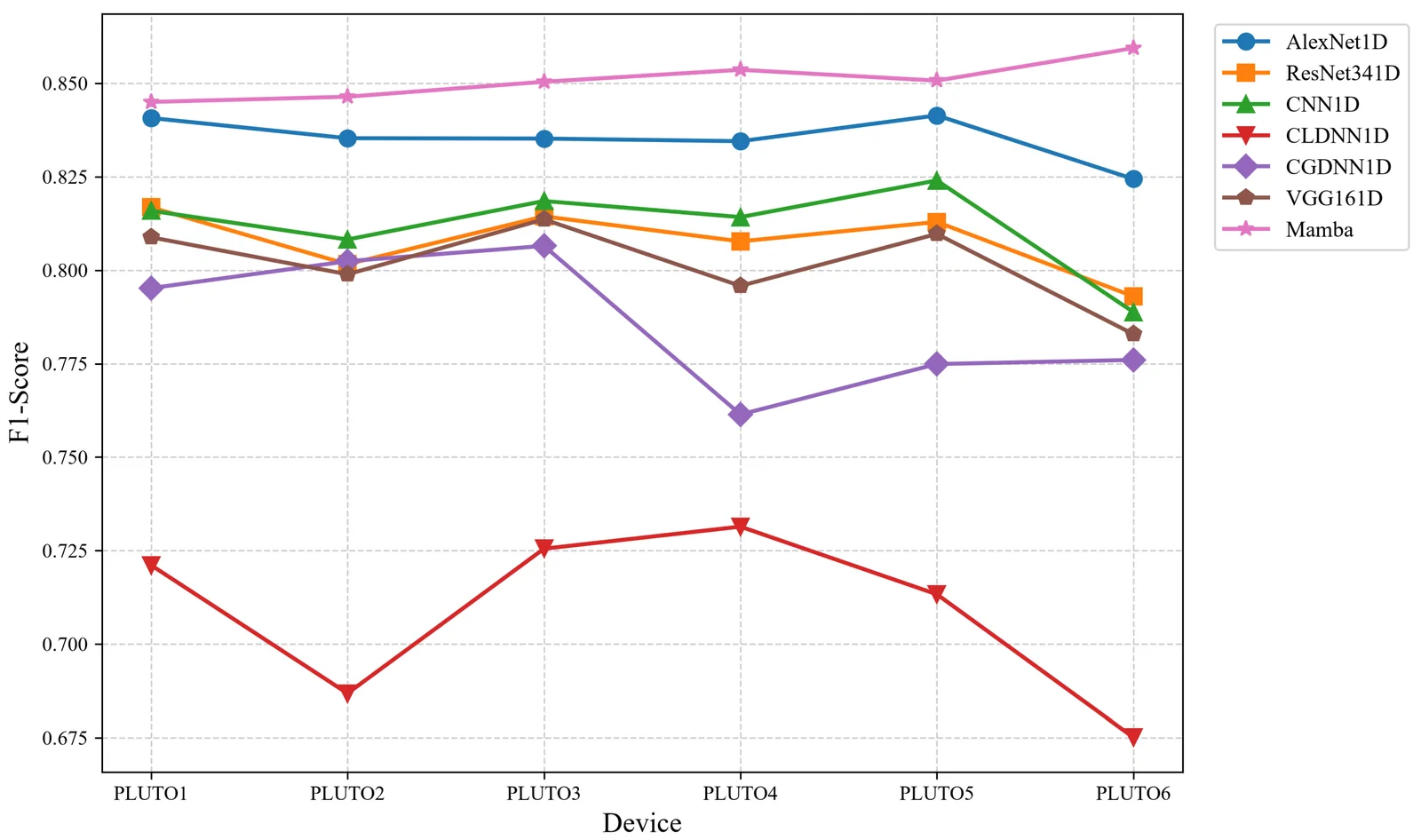
The F1-Score of the seven benchmark models on different receivers.

Figure 9, Figure 10, Figure 11 and Figure 12 present the accuracy, precision, recall, and F1-score, respectively, of the seven benchmark models across the six receivers. The results reveal different levels of sensitivity to receiver variations among the network architectures. Mamba, AlexNet, and ResNet34 exhibit relatively consistent performance across all six receivers, with only minor receiver-to-receiver fluctuations. In contrast, the hybrid architectures, particularly CLDNN and CGDNN, show more pronounced performance variations across different receivers.

These differences may be related to the ability of each model to disentangle transmitter-specific radio-frequency fingerprint features from receiver- and channel-dependent distortions. In practical acquisition scenarios, the received I/Q signals contain not only the intrinsic RF fingerprint characteristics introduced by the satellite transmitter, but also hardware-dependent impairments introduced by the receiving chain. Models with relatively stable performance across receivers may therefore be more effective at suppressing receiver-specific variations and extracting transmitter-related fingerprint representations. By contrast, models exhibiting larger cross-receiver fluctuations may be more sensitive to receiver-dependent components, such as front-end distortions and transient noise, which can be inadvertently learned as discriminative features and consequently reduce cross-receiver robustness.

Considering its performance stability across the six physical receivers, ResNet34 was selected as the baseline model for the subsequent experiments. Although ResNet34 did not achieve the highest overall accuracy, it is a well-established deep residual architecture with a moderate model size, relatively small performance variations across different receivers, and a network configuration consistent with the benchmark settings adopted in previous studies in this field. Based on these considerations, ResNet34 was used as the unified reference model in the subsequent SNR and input-sequence-length experiments to ensure the comparability and reproducibility of the experimental results.

### 5.3. Signal Length Experiment

ResNet34 was trained and tested with I/Q sequence lengths of 3000, 2048, 1024, and 512. For lengths below 3000, each subsequence was extracted from the original 3000-sample QPSK segment using a random starting index. Table 6 reports average performance across receivers.

As the input sequence length decreases from 3000 to 512 sampling points, the classification performance of ResNet34 declines significantly, with the accuracy decreasing from 0.8154 to 0.0213. When the sequence length is reduced from 3000 to 2048 sampling points, the model still maintains an accuracy of approximately 0.75. However, when the observation window is further shortened to 1024 sampling points or fewer, the recognition performance drops sharply, indicating that short observation sequences cannot provide sufficient and stable discriminative information for satellite identification. This phenomenon may be related to the temporal distribution characteristics of satellite RF fingerprints. Under complex and low-SNR space-channel conditions, subtle individual differences introduced by transmitter hardware imperfections generally require a sufficiently long observation window to accumulate and form stable representations. Short input sequences contain limited effective fingerprint information, while random cropping further increases the uncertainty in the coverage of discriminative features among different samples, making it difficult for the network to learn stable and distinguishable RF fingerprint representations. Based on these results, signal sequences with a length of 3000 sampling points are used as the input in the subsequent experiments to ensure reliable identification performance and experimental results.

**Figure 13:**
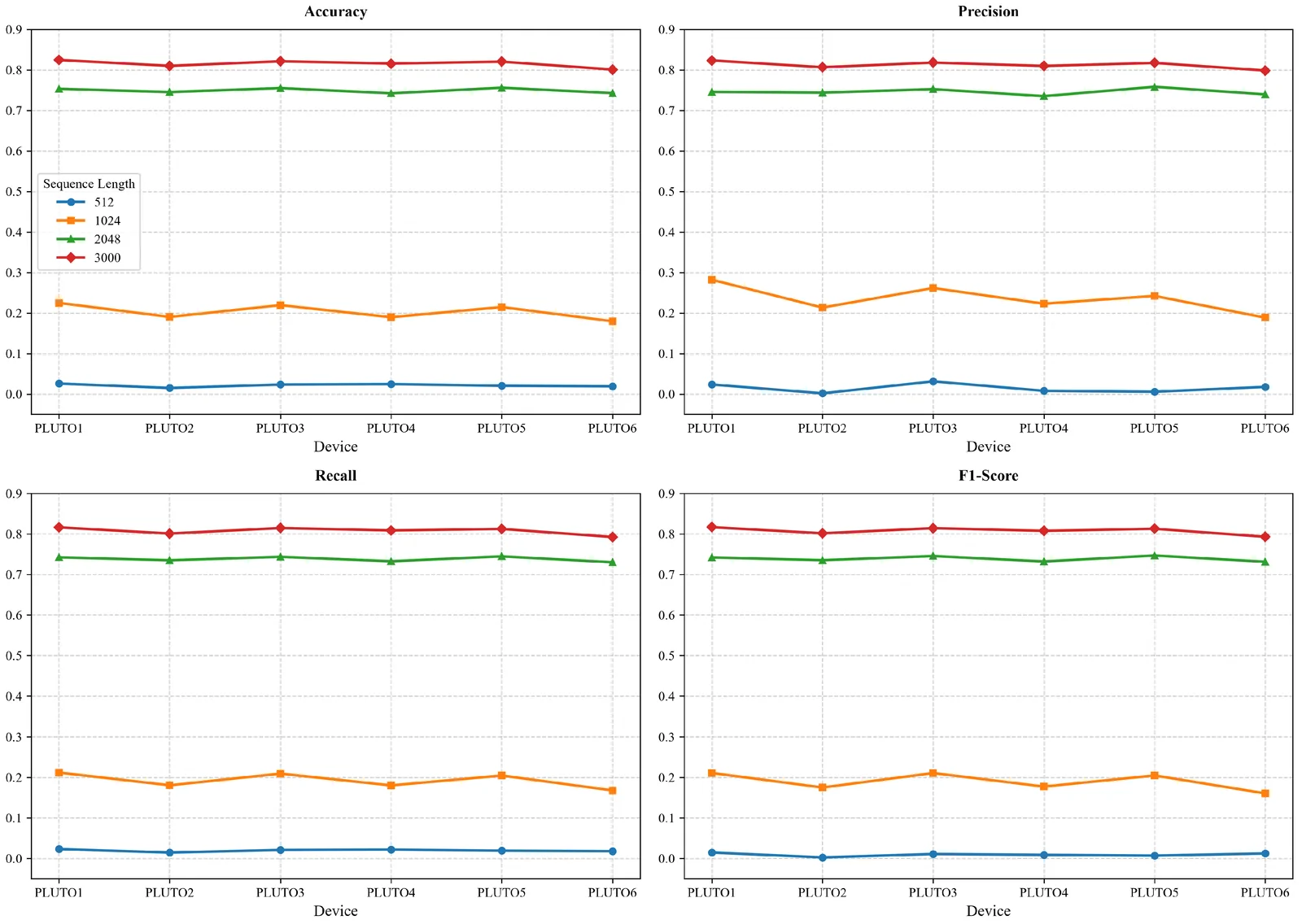
Recognition performance of different receivers at various lengths.

Figure 13 presents the identification performance of the six receivers under different input sequence lengths. With sufficiently long observation windows, namely 3000 and 2048 sampling points, the ResNet34 model exhibits highly consistent performance across the six physical receivers, from PLUTO1 to PLUTO6. As shown by the performance curves, the evaluation metrics remain relatively stable within these effective sequence-length ranges, with only small receiver-to-receiver variations. These results indicate that, given a sufficiently long observation window, the model can maintain stable identification performance across different receivers, and the hardware differences among the six physical receivers have a relatively limited influence on the overall identification results.

### 5.4. Signal-to-Noise Ratio Experiment

For SNR validation, a separate ResNet34 classifier was trained for each receiver using 3000-sample inputs. Test samples were grouped into 2 dB SNR intervals. The number of test samples for each receiver is listed in Table 7.

**Figure 14:**
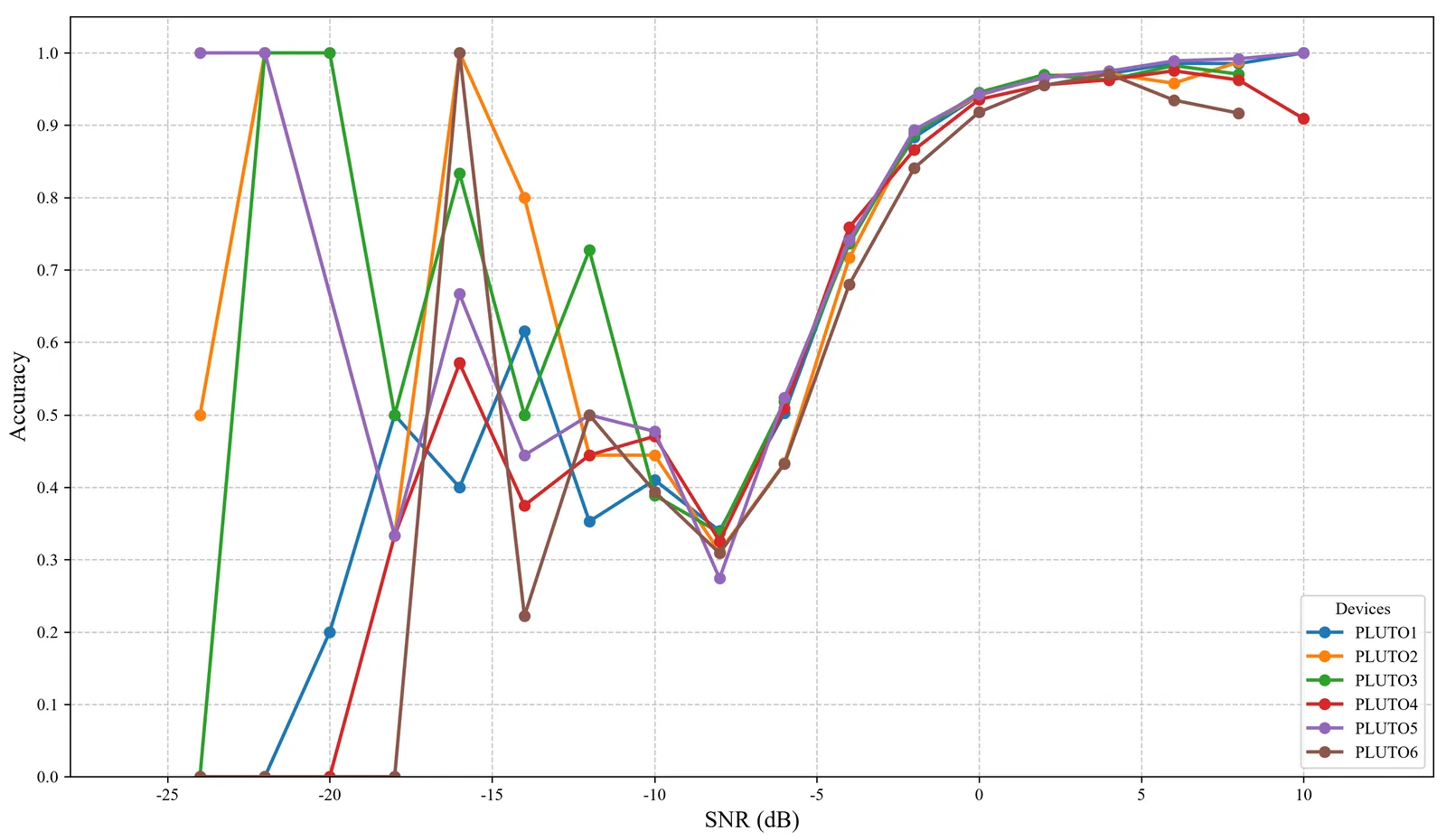
Signal-to-noise ratio experimental results.

The SNR experimental results are shown in Figure 14. It should be noted that, at SNR values of −8dB and below, extremely low-SNR samples are naturally scarce in the measured satellite-to-ground signals, resulting in insufficient numbers of test samples within these intervals. Consequently, the estimated recognition accuracies exhibit substantial fluctuations, with the accuracy of some receivers dropping to nearly zero. Because these results are strongly affected by the limited sample size and therefore lack sufficient statistical reliability, the SNR intervals at −8dB and below are excluded from the subsequent performance analysis.

Within the better-populated SNR range above −8dB and up to 10dB, the recognition accuracies of all six receivers increase consistently with SNR. Under an SNR of −4dB, the model maintains a mean accuracy of approximately 72.95%. When the SNR exceeds 0dB, the mean recognition accuracy increases to approximately 93.74%, and the performance approaches its maximum and remains relatively stable at SNR values of approximately 5dB and above. This behavior is consistent with the physical characteristics of RF fingerprints. Transmitter-specific fingerprints are typically manifested as subtle hardware-induced distortions embedded in the received waveform. Under low-SNR conditions, these weak discriminative components can be masked by background noise, causing the features extracted by the front-end convolutional layers to be dominated by noise-related variations. As the SNR increases, the transmitter-related fingerprint components become more distinguishable from the noise background, enabling the network to extract more stable and discriminative representations and thereby improving identification accuracy.

The IridiumRF-MR dataset contains samples covering a wide range of SNR conditions and can provide data support for the development, training, and evaluation of neural-network-based satellite identification methods under low-SNR environments.

### 5.5. Cross-Receiver Experiment

To further evaluate the cross-receiver usability of IridiumRF-MR, a single-source cross-receiver identification experiment was conducted. PLUTO1 was selected as the source receiver, and ResNet34 was used as the baseline classifier. The model was trained only using the training samples from PLUTO1. All model parameters were fixed, and no fine-tuning or parameter adaptation was performed using data from the other receivers.

The trained PLUTO1 model was first evaluated on the held-out PLUTO1 test subset to provide an intra-receiver reference. The same fixed model was then directly evaluated on the corresponding test subsets of PLUTO2, PLUTO3, PLUTO4, PLUTO5, and PLUTO6. Therefore, the PLUTO1 → PLUTO1 result represents the intra-receiver baseline, whereas PLUTO1 → PLUTO2–PLUTO6 represent cross-receiver evaluations. Accuracy, precision, recall, and F1-score were used as the evaluation metrics.

Table 8 presents the cross-receiver identification results. When the model was trained and tested on PLUTO1, the recognition accuracy was 0.8244, providing the intra-receiver reference. Without any retraining or fine-tuning, the same model achieved accuracies of 0.8356, 0.8406, 0.8403, 0.8393, and 0.8193 on PLUTO2–PLUTO6, respectively. The corresponding F1-scores ranged from 0.8136 to 0.8366. The average accuracy over the five cross-receiver test sets was 0.8350, which was comparable to the intra-receiver result.

These results indicate that the satellite-dependent discriminative features learned from PLUTO1 can be transferred directly to the other physical receivers of the same hardware model under the controlled common-input acquisition configuration. Although device-to-device hardware differences exist among the six independent receiving chains, no substantial degradation was observed in this single-source cross-receiver evaluation. This result supports the use of IridiumRF-MR for studying receiver-domain effects and cross-receiver satellite identification.

## 6. User Notes

The dataset can be used for individual satellite identification, cross-receiver evaluation, SNR-related studies, input-length analysis, and identification under different modulation types.

The source data are stored in MATLAB format. The I/Q signals are stored as two-channel real-valued arrays, where the last dimension contains the in-phase (I) and quadrature (Q) components, respectively. For neural networks that require real-valued inputs, the recommended representation is a two-channel tensor [I;Q] with shape L×2.

Before training, users should verify that every numeric label maps to one and only one satellite identifier, and that I/Q arrays and metadata have equal sample counts. Because very-low-SNR intervals are sparsely populated, accuracy should be accompanied by the number of samples and, preferably, a confidence interval.

## 7. Conclusions and Future Work

This study addresses the lack of data support for satellite physical-layer security authentication by constructing and validating a one-dimensional I/Q radio-frequency fingerprint dataset of Iridium satellites. During data collection, six SDR receivers of the same model were used to continuously acquire signals for approximately 72 h, capturing real Iridium downlink signals under satellite-to-ground channel conditions with varying Doppler shifts and environmental noise. The SNR of the collected samples ranges from −15 to 10dB. The dataset covers 66 Iridium satellites and contains 367,658 receiver-level valid samples. Each sample preserves the main burst components, including the unmodulated tone, BPSK field, and QPSK data segment. Satellite identities are assigned under non-cooperative conditions by jointly matching acquisition timestamps, measured Doppler shifts, and theoretical orbital information. In addition, each sample is associated with metadata such as satellite identity, SNR and timestamp, while the number of samples across different satellite classes remains relatively balanced.

The effectiveness of the dataset for individual satellite identification was validated through multi-model comparisons, input-sequence-length experiments, SNR experiments and cross-receiver experiment. The input-sequence-length experiments demonstrate that sufficiently long observation sequences are beneficial for obtaining stable RF fingerprint representations and provide a reference for selecting the input signal length in specific emitter identification of satellites. The SNR experiments further reveal the influence of signal quality on RF fingerprint recognition performance and provide a data basis for feature extraction and recognition-model design under low-SNR conditions. In the cross-receiver experiment, the model trained on PLUTO1 achieved comparable performance when directly evaluated on PLUTO2–PLUTO6 without fine-tuning, indicating limited receiver-domain degradation among the same-model receivers under the controlled common-input acquisition configuration.

Several issues remain to be further investigated. In practical applications, receiving devices are often produced by different manufacturers and may use different hardware models, resulting in more pronounced differences in RF front-end characteristics. Since the experiments in this study were mainly conducted using receivers of the same model, the evaluation of cross-device generalization remains limited. Future work should therefore collect data using receivers from different manufacturers and of different models, and investigate domain adaptation and domain generalization methods to improve model robustness against receiver-dependent variations. In addition, low-SNR conditions, different observation periods and complex channel environments may all affect the stability of RF fingerprint features. Further research is therefore needed on robust feature extraction under low-SNR conditions, as well as cross-time and cross-channel identification, to improve the applicability of individual satellite identification methods in realistic and complex scenarios.

## 8. Specifications Table


**Subject**
Engineering.
**Specific subject area**
Satellite radio-frequency fingerprinting, individual satellite identification, and low Earth orbit satellite signal processing.
**Type of data**
One-dimensional complex I/Q signal sequences and associated metadata stored in MATLAB MAT-files. The signal data include processed unmodulated tone, BPSK, and QPSK components.
**Data collection**
Iridium downlink signals were continuously collected for approximately 72 h using six ADALM-PLUTO software-defined radio receivers of the same model. The receivers were connected to a common antenna signal through a one-to-six power divider and operated with identical acquisition parameters. The center frequency was 1626.25MHz, the sampling rate was 960kHz, and the receiver gain was 50dB. An external 10MHz reference clock was used to improve frequency and sampling consistency among the receivers.
**Data source location**
Xi’an, Shaanxi Province, China, approximately 108.9139° E and 34.2361° N.
**Data accessibility**
Repository name: Zenodo. Data identification number: DOI: 10.5281/zenodo.21926940. Direct URL to data: https://doi.org/10.5281/zenodo.21926940. Instructions for accessing these data: The dataset is publicly available through Zenodo under the Creative Commons Attribution 4.0 International (CC BY 4.0) license.
**Related research article**
None.
**Previously published dataset or data descriptor**
None.

## Figures and Tables

**Table 1:** Variables and data organization in data_pluto1.mat.

Variable	Content	Format
BPSK	BPSK modulation I/Q data	63,407 × 461 × 2, single
Doppler	Measured Doppler frequency shift	1 × 63,407, single, unit: Hz
Latitude	Collection site latitude	1 × 63,407, single
Longitude	Collection site longitude	1 × 63,407, single
QPSK	QPSK data-segment I/Q data	63,407 × 3000 × 2, single
Solar	Solar and geomagnetic activity	63,407 × 4, cell
Time	Sample collection timestamp	63,407 × 1, cell
Weather	Weather, temperature, and humidity	63,407 × 3, cell
pilot	Unmodulated single-frequency I/Q data	63,407 × 2496 × 2, single
satlabel	Iridium category label	1 × 63,407, uint8, range: 0–65
satname	Satellite name and catalog number	63,407 × 1, cell
snr	Signal-to-noise ratio	1 × 63,407, single, unit: dB

**Table 2:** Receiver-level valid observation counts for the six receivers.

Receiver	Valid Samples	Satellite Classes
PLUTO1	63,407	66
PLUTO2	60,406	66
PLUTO3	61,467	66
PLUTO4	62,547	66
PLUTO5	61,990	66
PLUTO6	57,841	66
**Total**	**367,658**	**66**

**Table 3:** Principal acquisition settings.

Parameter	Value
Center frequency	1626.25 M Hz
Sampling rate	960 k$\mathrm{sample}\;s^{-1}$
Receiver gain	50 dB
Samples per captured frame	9600 complex samples
External clock frequency	10 MHz
Number of receivers	6
Acquisition duration	approximately 72 h

**Table 4:** Excerpt of the Iridium satellite identifier-to-class mapping.

Iridium Satellite Identifier	Dataset Label
IRIDIUM_120_42805	0
IRIDIUM_132_42961	1
IRIDIUM_119_42959	2
IRIDIUM_133_42955	3
IRIDIUM_130_43073	4
IRIDIUM_104_41922	5
IRIDIUM_110_43481	6
IRIDIUM_154_43574	7
IRIDIUM_156_43576	8

**Table 5:** Average recognition performance of the seven baseline models across the six receivers.

Model	Accuracy	Precision	Recall	F1-Score
AlexNet	0.8406	0.8379	0.8348	0.8353
ResNet34	0.8154	0.8122	0.8076	0.8077
CNN	0.8172	0.8155	0.8106	0.8116
CLDNN	0.7160	0.7249	0.7116	0.7089
CGDNN	0.7904	0.7997	0.7845	0.7861
VGG16	0.8094	0.8078	0.8003	0.8016
Mamba	0.8567	0.8521	0.8521	0.8509

**Table 6:** Average ResNet34 performance for different input sequence lengths.

Length	Accuracy	Precision	Recall	F1-Score
3000	0.8154	0.8122	0.8076	0.8077
2048	0.7490	0.7458	0.7381	0.7388
1024	0.2030	0.2350	0.1924	0.1899
512	0.0213	0.0145	0.0196	0.0093

**Table 7:** Number of test samples used in the receiver-specific SNR analysis.

Receiver	Test Samples
PLUTO1	6340
PLUTO2	6040
PLUTO3	6146
PLUTO4	6254
PLUTO5	6199
PLUTO6	5784

**Table 8:** Cross-receiver identification performance using PLUTO1 as the source receiver.

Test Receiver	Accuracy	Precision	Recall	F1-Score
PLUTO1	0.8244	0.8233	0.8163	0.8168
PLUTO2	0.8356	0.8351	0.8291	0.8308
PLUTO3	0.8406	0.8406	0.8347	0.8366
PLUTO4	0.8403	0.8403	0.8337	0.8358
PLUTO5	0.8393	0.8383	0.8328	0.8346
PLUTO6	0.8193	0.8184	0.8117	0.8136

## Data Availability

The IridiumRF-MR dataset is publicly available in Zenodo at https://doi.org/10.5281/zenodo.21926940.

## Abbreviations

The following abbreviations are used in this manuscript:

BPSK	Binary phase-shift keying
CFAR	Constant false alarm rate
I/Q	In-phase/quadrature
LEO	Low Earth orbit
LNA	Low-noise amplifier
QPSK	Quadrature phase-shift keying
RF	Radio frequency
SDR	Software-defined radio
SEI	Specific emitter identification
SNR	Signal-to-noise ratio
STK	Satellite Tool Kit
TLE	Two-line element

## References

[1] Zheng J.K., Luan T.H., Li G.J., Zhao J.W., Yin Z.S., Cheng N., Pan J. (2026). Low Earth Orbit Satellite Networks: Architecture, Key Technologies, Measurement, and Open Issues. IEEE Netw..

[2] Cao J., Sun Y., Zhang Y.Y., Ma R.H., Zhang T.T., Su Z., Su P.R., Li H., Feng D.G. (2026). A Survey of Security in Satellite Internet Networks. Sci. Sin. Inf..

[3] Liu Z., Liu Z., Ruan G.X. (2022). A Radar Emitter Fingerprint Feature Resistant to Receiver Distortion. Proceedings of the 2022 IEEE 5th Advanced Information Management, Communicates, Electronic and Automation Control Conference (IMCEC).

[4] Tan Z., Qin H., Cong L., Zhao C. (2019). New Method for Positioning Using IRIDIUM Satellite Signals of Opportunity. IEEE Access.

[5] Florio A., Bnilam N., Talarico C., Crosta P., Avitabile G., Coviello G. (2024). LEO-Based Coarse Positioning Through Angle-of-Arrival Estimation of Signals of Opportunity. IEEE Access.

[6] Oligeri G., Sciancalepore S., Raponi S., Di Pietro R. (2023). PAST-AI: Physical-Layer Authentication of Satellite Transmitters via Deep Learning. IEEE Trans. Inf. Forensics Secur..

[7] Solenthaler C., Smailes J., Strohmeier M. OrbID: Identifying Orbcomm Satellite RF Fingerprints. Proceedings of the 3rd Workshop on Security of Space and Satellite Systems (SpaceSec).

[8] Qin H., Tan Z., Cong L., Zhao C. (2019). Positioning Technology Based on Iridium Signals of Opportunity. J. Beijing Univ. Aeronaut. Astronaut..

[9] Krizhevsky A., Sutskever I., Hinton G.E. (2017). ImageNet Classification with Deep Convolutional Neural Networks. Commun. ACM.

[10] He K., Zhang X., Ren S., Sun J. Deep Residual Learning for Image Recognition. Proceedings of the 2016 IEEE Conference on Computer Vision and Pattern Recognition (CVPR).

[11] Sainath T.N., Vinyals O., Senior A., Sak H. Convolutional, Long Short-Term Memory, Fully Connected Deep Neural Networks. Proceedings of the 2015 IEEE International Conference on Acoustics, Speech and Signal Processing (ICASSP).

[12] Hu P., Yang W., Pu N., Peng Y., Ding X. A New Deep Architecture for Digital Signal Modulation Classification over Rician Fading. Proceedings of the 2021 IEEE 6th International Conference on Computer and Communication Systems (ICCCS).

[13] Simonyan K., Zisserman A. (2014). Very Deep Convolutional Networks for Large-Scale Image Recognition. arXiv.

[14] Gu A., Dao T. (2023). Mamba: Linear-Time Sequence Modeling with Selective State Spaces. arXiv.

